# A Tensile Test-Based Investigation of Load Deflection Properties of Three Commercial Superelastic Nickel-Titanium Orthodontic Archwires: An In Vitro Study

**DOI:** 10.7759/cureus.114032

**Published:** 2026-08-05

**Authors:** Ravisankar Vijayan, Latheef Vadakkepediyakkal, Jayan James

**Affiliations:** 1 Orthodontics and Dentofacial Orthopaedics, Government Dental College, Thrissur, Thrissur, IND; 2 Orthodontics and Dentofacial Orthopaedics, Government Dental College, Kozhikode, Kozhikode, IND; 3 Orthodontics and Dentofacial Orthopaedics, Government Dental College, Kottayam, Kottayam, IND

**Keywords:** alignment, load deflection rate, mechanical properties, nickel titanium archwires, super-elastic wires

## Abstract

Introduction: Nickel-titanium (Ni-Ti) alloys are widely used during the initial alignment phase of fixed orthodontic treatment because of their ability to deliver light and relatively constant forces over a wide range of activation. This study compared the force values at predetermined deflections of three commercially available super-elastic Ni-Ti archwire brands: 3M Unitek (Solventum Orthodontics Corporation, Monrovia, California, United States), G&H (G&H Orthodontics, Franklin, Indiana, United States), and DTech (D-Tech Dental Technologies, Pune, Maharashtra, India).

Materials and methods: Preformed straight length 0.019 × 0.025-inch super-elastic Ni-Ti archwires from three manufacturers were evaluated. Specimens were divided into three groups: Group I (3M Unitek), Group II (G&H), and Group III (D-Tech). A three-point bending test was performed to assess load-deflection characteristics. Load values were recorded at 0.5 mm and 1.0 mm deflection during loading and at 0.5 mm deflection during unloading. Intergroup comparisons were performed using appropriate statistical tests.

Results: A statistically significant difference among the three brands was observed at 0.5 mm loading. Group III demonstrated the highest mean rank, followed by Group II and Group I, indicating higher loading forces at this level of deflection. No statistically significant differences were found among the groups at 1.0 mm loading or 0.5 mm unloading. Pairwise analysis revealed a significant difference between Groups III and I, whereas differences between Groups III and II and between Groups II and I were not significant.

Conclusion: Differences in load-deflection behaviour were observed among the tested archwires, particularly at 0.5 mm loading. Group III exhibited higher loading-force values than Group II and Group I, with Group III showing the lowest, while no significant differences were identified at 1.0 mm loading or 0.5 mm unloading. These findings indicate variations in mechanical performance among commercially available super-elastic Ni-Ti archwires; however, clinical trials are required before drawing conclusions regarding clinical performance or superiority.

## Introduction

Nickel-titanium (Ni-Ti) alloys are widely regarded as the material of choice for the initial alignment phase of fixed orthodontic treatment because of their ability to deliver light and relatively constant forces over a wide range of activation [1]. The alloy was originally developed in the late 1960s by Buehler for an aerospace program in Silver Springs, Maryland [2,3]. Subsequently, during the 1970s, Andreasen, in collaboration with the Unitek Company, introduced Ni-Ti alloys into orthodontics under the name Nitinol, an acronym of Nickel Titanium Naval Ordnance Laboratory [4].

The distinctive mechanical properties of superelastic Ni-Ti wires are primarily due to their ability to undergo a reversible martensitic phase transformation at or near room temperature [5]. In these alloys, the austenitic phase, characterized by a body-centered cubic crystal structure, predominates under high temperature and lower stress and exhibits greater stiffness. When stress is applied during engagement into malaligned teeth, the alloy transforms into the martensitic phase, which possesses a more stable hexagonal crystal structure and is more deformable.

Kusy classified commercially available Ni-Ti archwires into three categories: martensitic stable, martensitic active, and austenitic active alloys [5]. Martensitic stable wires, such as Nitinol, are extensively work-hardened Ni-Ti alloys in which the martensitic phase remains stable, and no phase transformation occurs. Although these wires exhibit greater stiffness compared to other types, they still have approximately one-fifth that of stainless-steel wires. Martensitic active Ni-Ti wires, including Neo Sentalloy (Dentsply Sirona, Charlotte, North Carolina, United States), Bioforce Sentalloy (Dentsply Sirona), and Copper Niti™ (Ormco Corporation, Brea, California, United States), are less cold-worked Ni-Ti wires and therefore undergo martensitic transformation thermally. These thermoelastic wires exhibit shape memory properties and regain their original shape upon temperature changes. Austenitic active wires, such as Japanese Ni-Ti and Chinese Ni-Ti, demonstrate super-elastic behavior. During clinical activation, stress generated by archwire deflection induces transformation of the austenitic phase into martensite during loading, while the reverse transformation occurs during unloading. Unlike martensitic stable wires, these super-elastic alloys exhibit a plateau region in the load-deflection curve, where substantial strain occurs with the minimal increase in stress. This property enables the wire to deliver relatively constant forces over a wide range of deflection, making super-elastic Ni-Ti archwires particularly advantageous during the alignment stages [5].

The market is flooded with various brands of super-elastic Ni-Ti archwires, and manufacturers often do not explicitly disclose their material properties. Consequently, product selection is frequently influenced by local clinical preferences and practitioner curiosity regarding inter-manufacturer differences. Load deflection rate (LDR) is the deflection produced for a given load [6]. Superelastic Ni-Ti wires usually have much less LDR compared to Nitinol [5]. Clinical experience suggests that all brands of superelastic Ni-Ti do not perform identically, thereby influencing the final displacement pattern of the dentition. Therefore, the choice of archwire requires careful evaluation of the clinical requirements together with an appropriate balance between physical properties, cost-effectiveness, and material availability

Accurate evaluation of the material properties of orthodontic appliances has become increasingly important with the growing emphasis on analytical methods in appliance design. Because superelastic Ni-Ti archwires continue to be used extensively in contemporary orthodontic practice and are likely to remain integral to future treatment mechanics, the present study was undertaken to evaluate the force values generated by these archwires at predetermined deflections during three-point bending. The objective of this study was to compare the force values at fixed deflections of three commercially available super-elastic Ni-Ti archwire brands.

## Materials and methods

Preformed straight-length 0.019 × 0.025-inch super-elastic Ni-Ti archwires from three manufacturers, 3M Unitek (Lot No. N5994; Solventum Orthodontics Corporation, Monrovia, California, United States), G&H (Lot No. 390054; G&H Orthodontics (Franklin, Indiana, United States), and DTech (Lot No. 1221-1140; D-Tech Dental Technologies, Pune, Maharashtra, India), were included in the study. The study was approved by the Institutional Ethics Committee of Government Dental College Thrissur (study number: 016/IEC/GDCTSR/2022).

Sample size calculation

A pilot study was conducted to estimate the sample size. The samples were divided into three groups: Group I (3M), Group II (G&H), and Group III (D-Tech). All specimens within each group were obtained from the same manufacturing batch. Since three groups (3M Unitek, G&H Orthodontics, and D-Tech) were compared, the sample size was calculated based on the outcome variable requiring the largest sample. The highest sample size was obtained for the load deflection values at 0.5 mm loading (g) when comparing 3M Unitek and D-Tech. The calculation was performed using nMaster software (Department of Biostatistics, Christian Medical College, Vellore), using a mean difference of 445 g, standard deviations of 45 g and 420 g, a two-sided significance level (α) of 0.05, 80% power, and an effect size of 1.6680. The minimum required sample size was seven specimens per group. Accordingly, seven specimens were included in each group. The operator was blinded to group allocation.

Intervention

**Figure 1:**
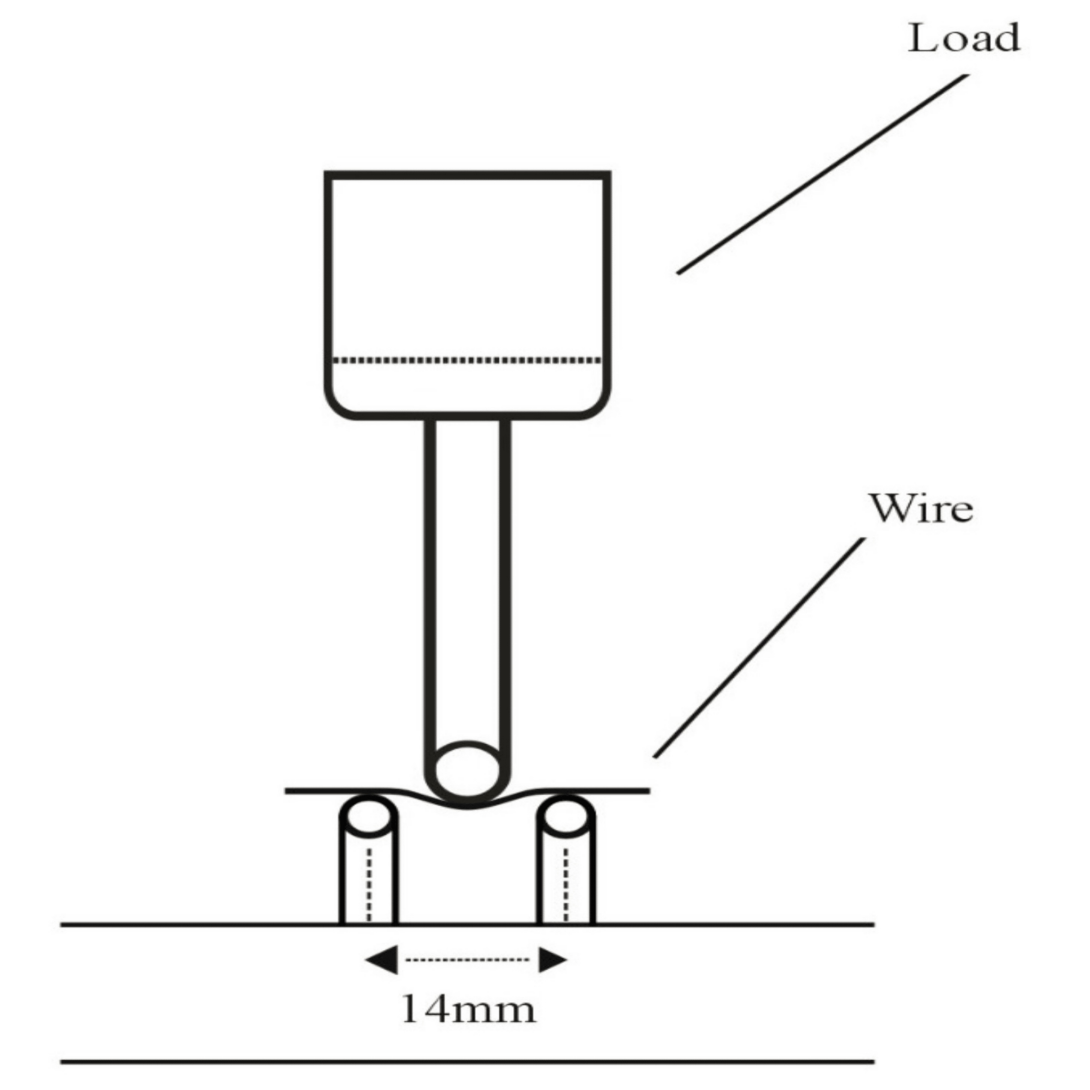
Set-up for three-point bend study Image Credit: Authors; created using Adobe Photoshop (Adobe Inc., San Jose, California, United States)

A three-point bend test was used to evaluate the load-deflection properties of specimens using a universal testing machine (Model No. N466605; Shimadzu Corporation, Kyoto, Japan) [7]. Prior to mechanical testing, all wire samples were examined for any surface imperfections and cut to a standardised length. A customised testing apparatus was fabricated for the procedure, consisting of two support poles placed at an inter-pole distance of 14 mm. The testing assembly consisted of two stainless-steel support poles positioned 14 mm apart. Standard edgewise brackets with a 0.022 × 0.028-inch slot were bonded to the supports, and each wire specimen was engaged into the bracket slots and secured using 0.012-inch stainless-steel ligatures to simulate the clinical bracket-wire interface. A centrally positioned loading rod of 2 mm diameter, attached to the lower crosshead of the testing machine, contacted the midpoint of the wire span and applied the bending load (Figure 1). The testing fixture was securely mounted on the universal testing machine, while the fixture itself was held firmly using mechanical wedge grips to prevent movement during testing. The wedge grips secured the testing assembly and did not directly grip the wire specimens, which were secured by ligatures.

All tests were performed at a controlled laboratory temperature of 37 ± 1°C under standard atmospheric conditions to simulate human body temperature. Before testing, the specimens were allowed to equilibrate to the testing temperature for five minutes. The universal testing machine was calibrated according to ISO (International Organization for Standardization) 7500-1 and ASTM (American Society for Testing and Materials) E4 standards, and the load cell was verified using certified reference standards by the deadweight master-cell comparison method before commencement of testing. 

Each specimen was subjected to deflection up to 1 mm at the centre of the span at a crosshead speed of 1 mm per min under a full-scale load setting of 100 N. Measurements were obtained during both the activation and deactivation phases of the test. The recorded parameters included load values at 0.5 mm deflection during loading, at 1 mm deflection during loading, and at 0.5 mm during unloading. All the wire segments were tested once each, and the machine software recorded the load continuously.

Statistical analysis

Data were entered into Microsoft Excel (Microsoft Corporation, Redmond, Washington, United States) and later analysed using IBM SPSS Statistics for Windows, version 25 (IBM Corp., Armonk, New York, United States). The normality of the data was assessed using the Shapiro-Wilk test. Intergroup comparisons of force values at predetermined deflections among the three arch wire brands were performed using the Kruskal-Wallis test. Post hoc pairwise comparisons for the significant variable were performed using the Mann-Whitney U test with Bonferroni correction (adjusted significance level p < 0.0167).

The normality of the mean values of the force values at predetermined deflections was assessed using the Shapiro-Wilk test. The p-values for deflection at 0.5 mm loading (p-value = 0.002), at 1.0 mm loading (p-value = 0.003), and at 0.5 mm unloading (p-value = 0.020) were all less than 0.05, indicating that the data were not normally distributed. Therefore, non-parametric statistical tests were used for data analyses.

## Results

The values increased from 0.5 mm loading to 1.0 mm loading, while unloading forces were lower than loading forces. The wider interquartile range (IQR) observed for 1.0 mm loading indicates greater dispersion of measurements compared with the other deflection characteristics.

**Figure 2:**
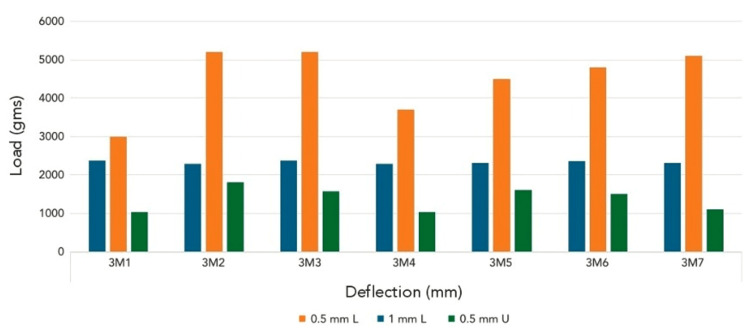
Load deflection of alloys from Group I (3M) L: loading; U: unloading; 3M: 3M Unitek (Solventum Orthodontics Corporation, Monrovia, California, United States)

**Figure 3:**
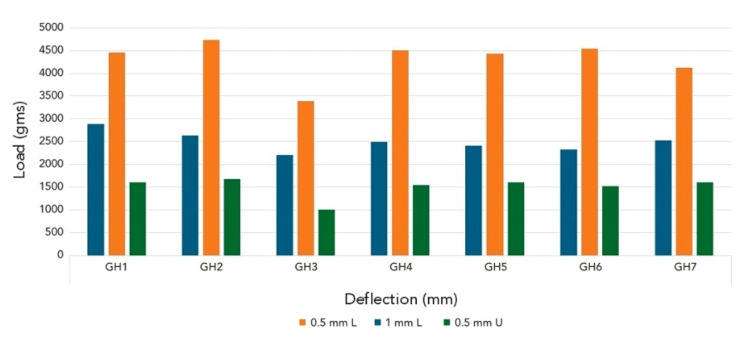
Load deflection of alloys from Group II (GH) L: loading; U: unloading; GH: G&H (G&H Orthodontics, Franklin, Indiana, United States)

**Figure 4:**
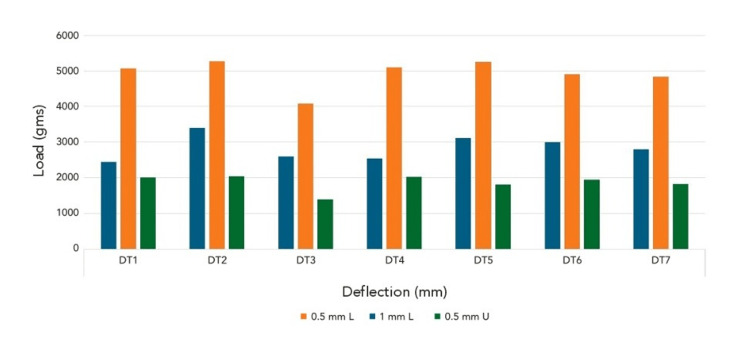
Load deflection of alloys from Group III (DT) L: loading, U: unloading; DT: D-Tech (D-Tech Dental Technologies, Pune, Maharashtra, India).

A statistically significant difference in load deflection was observed among the three brands at 0.5 mm loading (H = 7.288, p-value = 0.026). Group III exhibited the highest mean rank (14.43), followed by Group II (12.57) and Group I (6.00), indicating comparatively higher loading forces at this level of deflection. No statistically significant differences were observed among the brands at 1.0 mm loading (H = 2.589, p-value = 0.274) or 0.5 mm unloading (H = 5.429, p-value = 0.066), although Group III demonstrated the highest mean ranks for both parameters. Mean values of force at 0.5 mm loading, 1.0 mm loading, and 0.5 mm unloading for Groups I, II, and III are depicted in Figures 2-4, respectively. Comparisons of mean rank among the three groups are given in Table 1.

**Table 1:** Comparison of load deflection property among different Ni-Ti arch wire brands Kruskal–Wallis test applied for intergroup comparison, * Statistically significant at p value < 0.05. 3M: 3M Unitek (Solventum Corporation, Eagan, Minnesota, United States); GH: G&H Orthodontics (Franklin, Indiana, United States); DT: D-Tech (D-Tech Dental Technologies, Pune, Maharashtra, India); IQR: interquartile range; df: degree of freedom; Ni-Ti: nickel-titanium

Variable	3M (n = 7) Mean Rank	GH (n = 7) Mean Rank	DT (n = 7) Mean Rank	Overall Median (IQR)	Kruskal–Wallis H	df	p-value
Load Deflection at 0.5 mm Loading (g)	6.00	12.57	14.43	2440 (2380–2760)	7.288	2	0.026*
Load Deflection at 1.0 mm Loading (g)	10.86	8.43	13.71	4740 (3735–5200	2.589	2	0.274
Load Deflection at 0.5 mm Unloading (g)	8.57	9.00	15.43	1600 (1215–1905)	5.429	2	0.066

Following a significant Kruskal-Wallis test, post hoc pairwise comparisons were performed using the Mann-Whitney U test with Bonferroni correction. A statistically significant difference was observed between the III and I archwire groups (p = 0.001), whereas no significant differences were found between the III and II groups (p-value = 0.167) or the G&H and D-Tech groups (p-value = 0.948). The pairwise comparison of load deflection rate at 0.5 mm loading among them is depicted in Table 2 

**Table 2:** Pairwise comparison of load deflection at 0.5 mm loading among different Ni-Ti arch wire brands Post hoc pairwise comparisons were performed using the Mann–Whitney U test with Bonferroni correction following a significant Kruskal–Wallis test. * The Bonferroni-adjusted significance level was set at p-value < 0.0167. 3M: 3M Unitek (Solventum Corporation, Eagan, Minnesota, United States); GH: G&H Orthodontics (Franklin, Indiana, United States); DT: D-Tech (D-Tech Dental Technologies, Pune, Maharashtra, India); IQR: interquartile range; Ni-Ti: nickel-titanium

Comparison	Group	Median (IQR)	Mean Rank	Mann–Whitney U	p-value
3M vs GH	3M (n = 7)	2380 (2300–2380)	6.00	14.0	0.167
	GH (n = 7)	2630 (2200–2890)	9.00		
3M vs DT	3M (n = 7)	2380 (2300–2380)	4.00	0.000	0.001*
	DT (n = 7)	2600 (2440–3400)	11.00		
GH vs DT	GH (n = 7)	2630 (2200–2890)	7.57	24.0	0.948
	DT (n = 7)	2600 (2440–3400)	7.43		

## Discussion

Several theories have been proposed to explain the response of periodontal tissues to orthodontic forces and the relationship between the magnitude of force applied and the resulting tooth movement. A widely accepted concept is that optimal tooth movement is achieved with light, continuous forces [8]. Such forces are generally considered to be those that remain below the capillary blood pressure within the periodontal ligament, approximately 20-25 g/cm² of root surface area. When excessive forces are applied, compression of the periodontal blood vessels may occur, leading to interruption of blood flow. This can result in tissue hyalinisation, necrosis, and subsequent bone resorption, thereby delaying efficient tooth movement [9].

Archwires with a higher LDR deliver greater forces during activation, which may be less favourable for periodontal tissues. Ni-Ti archwires are widely used during the alignment phase of orthodontic treatment because they are capable of producing light and continuous forces, which are biologically desirable. With the increasing availability of different commercially manufactured Ni-Ti archwires, evaluating and comparing their mechanical behaviour has become increasingly important. Therefore, the present study assessed the load deflection properties of three commonly used superelastic Ni-Ti archwires from 3M Unitek, G&H Orthodontics, and DTech using a three-point bending test.

The three-point bending test, performed according to ADA Specification No. 32, is widely regarded as the standard method for assessing the mechanical behaviour of orthodontic wires that do not contain precious metals. To improve the accuracy and consistency of testing, Hurst et al. later introduced specially designed grips for securing the wire specimens in the Instron testing machine [10]. Although the test configuration primarily stimulates occlusogingival displacement and does not directly replicate clinical tooth movement, it remains one of the most commonly used methods for evaluating wire performance [11]. The test provides valuable information regarding the physical and biomechanical characteristics of orthodontic wires, offers high reproducibility, and enables standardised comparisons among different studies, including both laboratory investigations and clinical trials [12,13].

In the present investigation, three types of orthodontic wires from different manufacturers, all measuring 0.019 x 0.025 inches, were evaluated using a bending test. The test generates two sets of measurements: loading values, which represent the force required to engage the wire into the bracket system, and unloading values, which indicate the force released by the archwire and transmitted to the teeth during deactivation [14].

We compared the loading values at 0.5 and 1 mm and also the unloading values at 0.5 mm between wires. A statistically significant difference in load deflection was observed among the three brands at 0.5 mm loading. D-Tech exhibited the highest values, followed by G&H and 3M Unitek, indicating comparatively higher loading forces at this level of deflection. No statistically significant differences were observed among the brands at 1.0 mm loading or 0.5 mm unloading, although D-Tech demonstrated the highest mean values for both parameters. A statistically significant difference was observed between the 3M Unitek and D-Tech arch wire groups, whereas no significant differences were found between the 3M Unitek and G&H groups or the G&H and D-Tech groups.

 In the present study, all tested archwires maintained their ability to generate positive forces throughout the deactivation phase, with no evidence of permanent deformation. These findings differ from those reported by Mallory et al., who observed a marked reduction in force delivery by Copper Ni-Ti during the initial 0.5 mm deactivation [6]. The authors suggested that this behaviour may be attributed to the combination of substantial wire deflection and insufficient thermal energy to enable complete transformation of the alloy back to its austenitic phase. As a result, the wire was unable to sustain positive force generation during deactivation.

Al-Haroni et al. found no significant differences in the unloading force values between the super-elastic archwire (NT3-SE) and the 250°C heat-activated archwire (Thermal Ti-D) [14]. Although the difference was not statistically significant, the superelastic wire produced greater force levels during deactivation.

The superior superelastic characteristics observed in larger rectangular wires in the current study corroborate the findings of Segner and Ibe [15]. In addition, Garrec et al. reported that larger rectangular archwires required the least force during loading when evaluated using a three-point bending test [16].

The findings of the present in vitro investigation should be interpreted with caution, as laboratory conditions cannot fully replicate the complexity of the clinical orthodontic environment. Although considerable efforts are made to simulate intraoral conditions, each experimental setup possesses inherent limitations that restrict the direct application of laboratory results to patient care. Consequently, the mechanical behaviour observed in vitro may not accurately reflect the performance of archwires during clinical treatment. Laboratory testing remains valuable for comparing the relative performance of different wire types; however, these findings should ideally be correlated with evidence from clinical studies. For this reason, several authors have suggested that greater importance be placed on the relative ranking of force levels produced by different archwires rather than on the absolute force values reported in grams [17-19].

Certain limitations of the three-point bending test should be acknowledged. Deflections exceeding 1.0 mm are generally encountered only in cases of severe crowding or marked tooth irregularities [15]. In such clinical situations, clinicians often prefer round or smaller rectangular Ni-Ti archwires, thereby reducing the clinical applicability of larger-deflection test results. Consequently, the forces generated intraorally may not reach the superelastic plateau, and force delivery may remain dependent on the degree of wire deflection. Furthermore, the magnitude of strain experienced by an archwire during clinical treatment cannot be accurately quantified [15]. Additional intraoral factors, particularly friction between the archwire and bracket system, further influence force expression. Friction tends to increase the effective force during activation while reducing the force available during deactivation, thereby altering the force system compared with that observed under laboratory conditions [6]. The absence of an a priori power analysis is acknowledged as a limitation of the study. The present study evaluated specimens obtained from a single manufacturing batch of each brand. Therefore, the findings may reflect variation within a single production lot rather than the overall consistency of the manufacturer's products. In addition, the actual wire dimensions, elemental composition, transformation temperatures, and surface characteristics were not independently verified, although these factors may contribute to differences in mechanical performance among the archwires. Finally, the study evaluated force values at predetermined deflections rather than presenting complete loading and unloading curves with analysis of the superelastic plateau and hysteresis characteristics. Although this approach is appropriate for a preliminary comparative investigation, it provides only a limited characterisation of the superelastic behaviour of the tested archwires.

## Conclusions

D-Tech exhibited the highest values, followed by G&H and 3M Unitek, indicating comparatively higher loading forces at the initial level of deflection, which was statistically significant. No statistically significant differences were observed among the brands on further loading and unloading, although D-Tech demonstrated the highest mean values for both parameters. A statistically significant difference was observed between the 3M Unitek and D-Tech arch wire groups, whereas no significant differences were found between the 3M Unitek and G&H groups or the G&H and D-Tech groups. On this basis, according to the results of this study, Group I (3M Unitek) archwires have more favourable load deflection behaviour observed under the specific laboratory conditions tested.
